# A holistic review of fatherhood in humans and non-human animals: mechanisms of fatherhood, effects on fathers themselves, and interactions of fathers with others

**DOI:** 10.3389/fnbeh.2026.1779750

**Published:** 2026-05-29

**Authors:** Karen L. Bales, Ana Finnerty-Haggerty, Joanna Y. Guan, Lauren Meckler, Jasmine Saenz, Meghan J. Sosnowski, Shirin Afrakhteh, Christina L. Hung, Rani A. Purewal, Stephanie L. Oliinyk, Felisa J. Carbajal, Olufemi Shakuur Nyabingi

**Affiliations:** 1 Department of Psychology, University of California, Davis, Davis, CA, United States; 2 Department of Neurobiology, Physiology, and Behavior, University of California, Davis, Davis, CA, United States; 3 California National Primate Research Center, University of California, Davis, Davis, CA, United States; 4 Center for Neuroscience, University of California, Davis, Davis, CA, United States

**Keywords:** father, medial preoptic area, paternal care, synchrony, testosterone

## Abstract

Fathers are often the forgotten parent in mammals; due to the necessity of lactation for the survival of infants, mothers and motherhood are by far the better studied sex (a rather unusual phenomenon among most topics). However, the study of fathers has experienced a recent surge in interest. Here we review literature from a neuroscience, endocrine, and psychological perspective, on the mechanisms of fatherhood and roles of fathers across species, including the ways that fathering affects mental health, interactions with others, and partner relationships in humans and animals. Limitations and fundamental gaps remaining in the literature on fatherhood are identified.

## 1 Introduction

Fathering is undergoing a surge in scientific and public interest. The fascinating, recently published book by Hrdy (2024), *Father Time*, posits that interactions with babies and mothers can fundamentally change men, positioning human fatherhood, and the neural and physiological changes associated with it, as a contextually flexible phenomenon. Few reviews attempt to integrate the animal and human literature on multiple aspects of fatherhood, including the neuroendocrine, social, relationship, and health aspects, in a holistic fashion. In this paper, we examine the various aspects of fathering that have been studied in humans and non-human animals, both biological and psychosocial, starting with what is often assumed to be the first step that male mammals must take on their way to fathering: suppressing aggression towards infants. We then cover the neural and hormonal basis for fathering, the effects of fathering on the father’s mental and physical health, the relationships between fathers and their co-parents as influenced by infants, and finally, behavioral synchrony between fathers and offspring. Finally, we focus on the remaining gaps in the literature and our fundamental understanding of the mechanisms of fatherhood.

## 2 Mechanisms underlying the expression of fathering

### 2.1 Suppressing infanticide

In mammals, males in many species show aggression towards young, which can ultimately result in infanticide (Lukas and Huchard, 2014). Some males from those same species may instead show parental behaviors, suggesting that two pathways may simultaneously exist: one that induces infanticidal behavior and one that suppresses infanticide and promotes paternal behavior. The transition from infanticidal to parental behaviors has been well-documented in male laboratory mice (Tachikawa et al., 2013; Amano et al., 2017). Virgin males display high levels of infanticidal behavior, while adult males cohabitating with pregnant females show less infanticidal behaviors during pregnancy and no infanticidal behaviors after birth (Tachikawa et al., 2013).

Research into this drastic shift in behaviors towards pups has focused on the vomeronasal pathway, presumably because pheromone signaling allows male mice to detect pups and females. Isogai et al., (2018) found that both pup shape and pheromone cues are necessary to induce infanticidal aggression toward pups. After pup exposure, virgin males (but not fathers) show an increase in expression of the immediate early gene c-Fos in the vomeronasal organ (VNO) (Tachikawa et al., 2013). Ablation of the VNO decreased infanticide in virgin males (Tachikawa et al., 2013). Genetic knockouts of receptors in the VNO results in a decrease in infanticide in virgin male mice. Wu et al. (2014) found that genetic ablation of transient receptor potential cation channel subfamily C member 2 (Trpc2), a VNO-specific ion channel, impairs vomeronasal signaling, and that knocking out the Trpc2 gene in virgin males reduces pup-directed aggression while increasing parental behaviors. Vmn2r65 is a binding site for a pheromone (hemoglobins) produced by both adult females and pups, has also been implicated in infanticide suppression, with Vmn2r65 positive and Vmn2r88 positive neurons in the VNO being activated after pup exposure in virgin males (Wu et al., 2014). A double knockout of these receptor types in the VNO results in differences in pup-directed behavior in virgin males, suggesting that both receptors are activated to induce infanticidal behavior. The VNO projects to the accessory olfactory bulb (AOB), which also shows increased activity after pup exposure in virgin males (Chen et al., 2019). The AOB sends projections to the medial amygdala (MeA), which has been shown also to mediate infanticidal behavior in virgin males. Activation of GABAergic neurons in the posterodorsal region of the medial amygdala (MeApd) leads to infanticidal behavior in virgin males. Together, this suggests that the VNO has downstream effects on the MeA, via the AOB, to induce infanticidal behavior in virgin males, which when inhibited induces parental behaviors.

The medial preoptic area (MPOA) is important for parenting in both males and females of various species (Akther et al., 2014; Kuroda et al., 2024). While mammalian literature generally attributes this similarity to secondary evolution by males utilizing substrates evolved for mothering (Horrell et al., 2019), a more expansive evolutionary viewpoint attributes the conserved role of the MPOA back to hypothalamic neurons involved in territory and egg guarding in early vertebrates (Kuroda et al., 2024). Within the MPOA, optogenetic inactivation of glutamatergic neurons and optogenetic activation of GABAergic neurons decreases infanticidal behavior (Zhang et al., 2021). Lesioning the central part of the MPOA (cMPOA) in virgin male mice results in an increase of infanticidal behavior, whereas activation results in decreased infanticidal behavior (Tsuneoka et al., 2015). The cMPOA has GABAergic projections to the rhomboid nucleus of the bed nuclei of stria terminalis (BNSTrh), so lesioning the cMPOA results in increased activity in the BNSTrh. Lesioning the BNSTrh results in the suppression of infanticidal behavior and an increase in activity of the cMPOA, suggesting these two regions are each influencing the activity of the other to induce or suppress infanticidal behavior.

The MPOA is a heterogenous region with various cell types, including galanin receptor (gal) and calcitonin receptor (calcr) neurons, which have been shown to be important in the suppression of infanticide. Chemogenetic inhibition of calcr+ neurons in the cMPOA of virgin males decreases infanticidal behavior (Yoshihara et al., 2021), while stimulation of oxytocin neurons in the paraventricular nucleus of hypothalamus (PVH OT+), which suppresses activity in MPOA calcr+ neurons (Inada et al., 2022), induces parenting in virgin male mice. This activation of PVH OT + neurons is mediated by the lateral hypothalamus (LHA). Not only do PVH OT + neurons suppress cMPOA calcr+ neurons, but they also suppress activity of Urocortin-3 (Ucn3) neurons in the hypothalamic perifornical area (PeFA). Ucn3 + neurons in the PeFA are active during pup-directed aggression, and their inhibition leads to the suppression of infanticide in virgin males (Autry et al., 2021). Ucn3 + PeFA neurons receive inhibitory input from PVH OT + neurons and GABAergic MPOA neurons, suggesting this is another region that is vital for the suppression of infanticide. MPOA galanin (gal) neurons are also important for the suppression of infanticide, specifically the gal+ neurons that project to the periaqueductal gray (PAG), which are mostly GABAergic (Kohl et al., 2018). Optogenetic activation of MPOA gal+ neurons that project to the PAG decreases infanticide in virgin males (Kohl et al., 2018). Together, these results suggest an intricate balance of excitation and inhibition within this circuit to suppress infanticide in virgin male mice.

**Figure 1:**
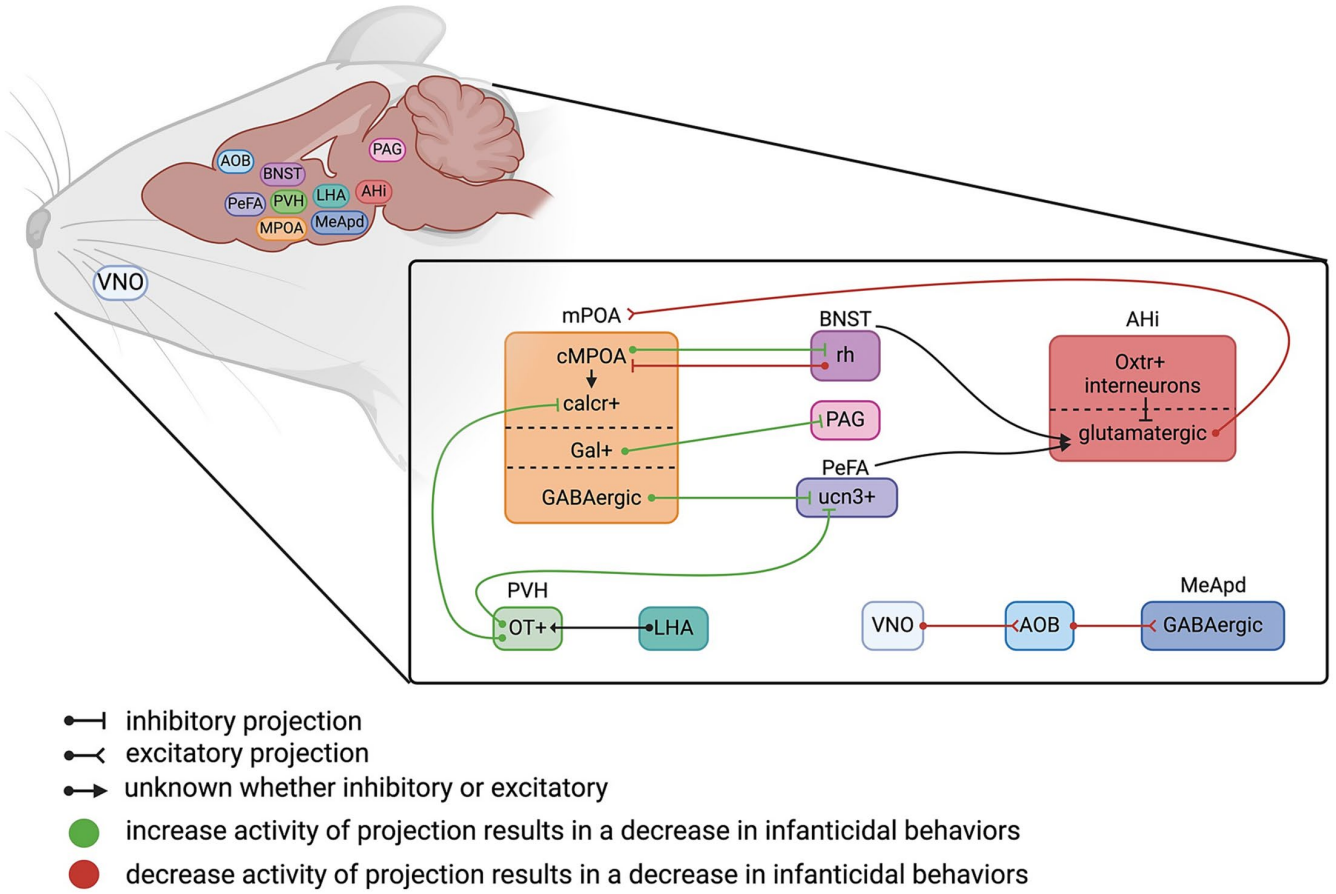
Neural circuits underlying the suppression of infanticide in laboratory mice. This figure summarizes the regions that have been implicated in the suppression of male infanticide. Medial preoptic area (mPOA); central medial preoptic area (cMPOA); calcitonin expressing neurons (calc+); galanin expression neurons (Gal+); GABA expressing neurons (GABAergic); bed nucleus of the stria terminalis (BNST); rhomboid nucleus of the bed nuclei of stria terminalis (rhBNST); periaqueductal gray (PAG); hypothalamic perifornical area (PeFA); urocortin-3 expressing neurons (ucn3+); amygdalohippocampal area (Ahi); oxytocin expressing neurons (Oxtr+/OT); paraventricular nucleus of hypothalamus (PVH); lateral hypothalamus (LHA); vomeronasal organ (VNO); accessory olfactory bulb (AOB); posterodorsal region of the medical amygdala (MeApd).

Another region that influences infanticidal behavior in mice and connects the MPOA, BNST, and PeFA is the amygdalohippocampal area (AHi). In mice, the AHi has glutamatergic projections to the MPOA, which receives input from both the BNST and PeFA (Sato et al., 2020). Activation of the AHi neurons that project to the MPOA increases infanticidal behavior in fathers. Infanticidal behavior is also increased in fathers-to-be males living with a female they have copulated with during the post-copulation (Sato et al., 2020). In this pre-parturition period, where they have not yet been exposed to their own pups, father-to-be males may still show some infanticidal behaviors. These infanticidal behaviors tend to decrease as parturition approaches and fathers-to-be become more parental, a trend that stimulation of MPOA-projecting neurons in the AHi disrupts. MPOA-projecting AHi neurons are inhibited by oxytocinergic neurons that synapse to oxytocin receptor positive interneurons in the AHi (Sato et al., 2020). This suggests a role for oxytocin release in the AHi in the suppression of infanticidal behaviors. Using male mice as a model, these studies show a complex system that regulates the transition from infanticidal to paternal behaviors (Figure 1).

### 2.2 Becoming a father – more than just suppressing infanticide

#### 2.2.1 Neural changes in animal fathers

##### 2.2.1.1 Neural change in animal fathers with transition to fatherhood

Parenthood is generally associated with a host of neurobiological changes in addition to those that suppress infanticide. For example, in California mice (*Peromyscus californicus*) and other rodents, exposure to pups changes neurogenesis, though the direction of this change depends on the time of testing (Horrell et al., 2021). In laboratory mice, paternal neurogenesis was associated with offspring recognition (Mak and Weiss, 2010). In rodents, many of the same important brain areas for parental care are shared across sexes, such as the MPOA, BNST, extended amygdala, hypothalamus, and olfactory bulb (Bales and Saltzman, 2016; Dulac et al., 2014; Kuroda et al., 2024; Rogers and Bales, 2019). This extends to parental care given to non-biological offspring (alloparenting), which is generally associated with oxytocin receptor expression in the nucleus accumbens (NAcc) (Kenkel et al., 2017). Many other mechanisms are similarly conserved across sexes, such as activation of the inferior frontal gyrus by infant cries in both mothers and fathers in humans (Piallini et al., 2015; Rogers and Bales, 2019).

Non-human animal studies offer the opportunity to interrogate the neuroendocrine changes associated with fatherhood and male care of infants in general. Biparental mandarin voles (*Microtus mandarinus*) display higher levels of oxytocin-immunoreactive neurons and more estrogen receptor alpha-immunoreactive neurons in the MPOA and BNST when comparing high-care fathers to low-care fathers (Li et al., 2015; Kuroda et al., 2024). In biparental Mongolian gerbils (*Meriones unguiculatus*), paternal care was associated with increased androgen receptor immunoreactivity in the MPOA, medial amygdala, and olfactory bulb, with the number of androgen receptor immunoreactive cells in gerbil fathers growing along with their pups (Martínez et al., 2019). For laboratory mice, galanin-expressing neurons in the MPOA are heavily associated with parental care across sexes (Moffitt et al., 2018; Wu et al., 2014) and are similarly linked in several related species of South American poison frog (Fischer et al., 2019). Finally, agouti-signaling protein has been identified as a possible new protein of interest for male care in African striped mice; expression of this protein is negatively associated with male care (Rogers et al., 2026).

Other genes also change expression with the onset of fatherhood or male care. A study of prairie vole (*Microtus ochrogaster*) males found that in the MPOA, genes related to synaptic plasticity were downregulated in fathers (Seelke et al., 2018). This downregulation parallels the decreased expression of genes associated with reduced dendritic branching and increased dendritic spine density in the MPOA of high-contact lactating rat dams (Parent et al., 2017), as well as the enhanced dendritic spine density of prefrontal cortex neurons in common marmoset fathers (Kozorovitskiy et al., 2006). These cross-species similarities suggest conserved neurobiological changes in synaptic plasticity related to parental experience. Such changes in gene expression similarly occur in non-mammalian species; for example, male three-spined stickleback fish (*Gasterosteus aculeatus*) experience distinct stages of fatherhood and parental care (Woolton, 2023), and a study examining gene expression across these stages found that in genes differentially expressed in parenthood, some were expressed across all stages while others were stage-specific (Bukhari et al., 2019). These gene expression patterns had similarities to the neurogenomic dynamics of mammalian female pregnancy and reproduction, with many orthologous genes shared between mouse maternal care and stickleback paternal care (Ray et al., 2016). Bukhari et al. (2019) found a link between transcription factors related to paternal care and those related to territorial aggression in sticklebacks, in which shared transcription factors were upregulated for one behavior and downregulated for the other. They drew possible parallels between the shared transcription factors for paternal care and territorial aggression in sticklebacks and how transcription factors modulate circuits for defensive behavior and paternal care in mammals.

##### 2.2.1.2 Neural correlates of fatherhood in animals, after the transition to first-time parenting

Studies of the neural basis for fatherhood over time, as well as mechanisms for specific, sustained behaviors such as carrying, infant grooming, or sharing food with infants, are much less studied in animals than the initial transition to fatherhood. However, there are a few exceptions. For common marmosets (*Callithrix jacchus*), a primate species where fathers provide a high level of paternal care (Ziegler, 2009), fatherhood is associated with increased dendritic spine density and a higher density of vasopressin V1a receptors in the prefrontal cortex, which declines as the infants get older (Kozorovitskiy et al., 2006). By contrast, in titi monkeys (*Plecturocebus cupreus*), pair-bonding primates where the male is the primary caregiver, parental experience in both males and females is associated with reduced V1a binding across multiple brain areas, with no significant influence of parenthood on frontal cortex V1a binding (Baxter et al., 2023). One notable exception found that intracerebroventricular OT increased tolerance for food transfer to offspring in common marmoset fathers (Saito and Nakamura, 2011). However, in general the neural basis for the maintenance of fathering behavior is not well studied in animal fathers.

#### 2.2.2 Neural changes in human fathers

##### 2.2.2.1 Transition to fatherhood in humans

A 2015 review of comparative research in the neurobiology of parenting suggested that the neural transition into parenthood differs between gestating mothers and non-gestating fathers (Feldman, 2015), despite the similar ending points. In gestating mothers, pregnancy hormones sensitize subcortical structures, priming the mother to care for the infant after birth. Non-gestating fathers lack the same hormonal sensitization and subsequently show less amygdala activation in response to infant cries than mothers, but consistently show mirror neuron activation, while mothers do not have consistent mirror neuron activation across studies (Feldman, 2015). This suggests that the path to fatherhood may be based more on experience with an infant, as opposed to the pregnancy hormone-sensitized structures in the gestating maternal brain (although hormonal changes are also present in the paternal brain; see the following section on endocrine changes in potential fathers).

It is becoming well-established that the transition to fatherhood leads to *structural* changes in the brains of human fathers, and research suggests, as noted above, that this may be associated with the experience of parenting and parental bonding. An early study which examined fathers at 2–4 weeks postpartum and 12–16 weeks postpartum found reductions in the orbitofrontal, posterior cingulate/precuneus, and insular cortices; however, they also found an increase in gray matter volume in the hypothalamus, amygdala, striatum, and lateral prefrontal cortex (Kim et al., 2014). Replicating the findings of Kim et al. (2014) of gray matter reduction in the precuneus, Paternina-Die et al. (2020) found that a greater reduction in this area was associated with a higher response to photos of their infants. Martínez-García et al. (2023) found that fathers show a significant reduction in gray matter across the entire cortex; this finding was stronger in men who felt that they were more strongly bonded to the infant (Saxbe and Martínez-García, 2024). Men with higher prenatal prolactin had greater reductions in the left posterior cingulate, left insula, and left nucleus accumbens (Aviv et al., 2023). The functionality of these structural changes is still an area that needs substantial study and may have effects on cognitive processes outside of the realm of infant care (see cognitive section 3.3.2).

There are also *functional* neural differences between fathers and non-fathers, some of them in the areas of structural changes detailed above. Expectant fathers with higher resting-state functional connectivity across the “mentalizing” network, specifically including the mPFC, the superior parietal lobule, and the lateral occipital cortex, showed higher father-child bonding as reported by the father following birth of the infant (Marshall et al., 2022). In comparison to their prenatal scans, first-time fathers show increased functional connectivity in the right lateral occipital lobe, bilateral temporal lobe, right angular gyrus, and thalamus (Vaccaro et al., 2026). During the transition to fatherhood, many of these cortical areas also show a higher change in activity in response to infant cues in fathers than they do in non-fathers [including many subareas of the frontal gyrus and the precentral gyrus (Rilling et al., 2026)]. In first-time fathers, greater activation of the left medial prefrontal cortex, left superior lateral occipital cortex and posterior cingulate gyrus were associated with higher observed caregiving sensitivity which coincided with decreases in amygdala activation in response to cry sounds (Thijssen et al., 2024). Conversely, new fathers exhibited increased amygdala connectivity with increased hours spent in direct childcare (Horstman et al., 2022).

In addition to cortical areas, the literature has implicated functional changes in reward pathways in the transition to fatherhood (Rilling et al., 2026). Fathers showed a larger increase than non-fathers in the response to infant stimuli in reward regions, including the caudate, putamen and ventral pallidum (all dopaminergic areas), among other regions (Rilling et al., 2026). In their response to infant cries, new fathers had widespread brain activation, notably including the mPFC, insular cortex, inferior frontal gyrus, the striatum, thalamus, auditory cortex, posterior cingulate, and dopaminergic midbrain areas such as the ventral tegmental area and substantia nigra (Li et al., 2018).

Activity in bilateral motor areas also change in first-time fathers between prenatal and postnatal scans (van’t Veer et al., 2019), with increases in response to situations that may be threatening to the infant. These changes may suggest a link between parental care and territory defense (Bukhari et al., 2019; Kuroda et al., 2024).

##### 2.2.2.2 Neural correlates of fatherhood in humans, after the transition to first-time parenting

The previously stated hypothesis that sex differences in the maternal and paternal brains are due to experience leads to several testable predictions. One study found that in same-sex male couples compared to different-sex couples, primary caregiver fathers had similar high amygdala activation as primary caregiver mothers and similar high superior temporal sulcus (STS) activation to secondary caregiver fathers (Abraham et al., 2014). While this paradigm has not been widely used, there is also one study that included both mothers and fathers in both same-sex and different-sex pairs; this study found no sex difference in attentional bias towards infant faces (Gemignani et al., 2024).

When comparing fMRI response to their own infant vs. a strange infant, fathers showed higher activity in many areas that have been previously implicated: the precuneus, posterior cingulate, orbitofrontal cortex, and inferior frontal gyrus (Newsome et al., 2025). Activity in the precuneus and posterior cingulate was positively correlated with father-child bonding and negatively correlated with parenting stress (Newsome et al., 2025). There was a significant difference between viewing photos of their infant and photos of their partner only in the precuneus (Newsome et al., 2025). Inhibition of response to infant cries has been linked to higher activity of prefrontal regulatory regions (Waizman et al., 2024).

Playing with one’s own child, vs. a virtual unfamiliar child in a “Cyberball” computer game, resulted in sex-specific differences in brain activation during inclusion and exclusion periods (Açıl et al., 2025). During inclusion, mothers demonstrated higher activity than fathers in several areas including postcentral gyrus, supplementary motor area, cerebellum, middle occipital gyrus, fusiform gyrus, putamen, and precuneus. During exclusion, fathers exhibited higher activity than mothers in intraparietal sulcus, cerebellum, extrastriate cortex, superior occipital gyrus, and middle cingulate gyrus. The authors interpreted these differences as mothers responding with more attention and reward areas during inclusion, and fathers responding with more mentalizing areas during exclusion (Açıl et al., 2025).

A bibliometric analysis of the literature on the neural basis for human parental care (Carollo et al., 2025), found that two of the most important reviews on this topic in fathers were (Sobral et al., 2022) and (Giannotti et al., 2022). One of these reviews suggested that paternal involvement may modulate the relationship between behavior and neurobiological responses in fathers (Giannotti et al., 2022), while the other called for the study of epigenetics and neuroplasticity in human fathers (Sobral et al., 2022). Overall, the evidence in human fathers suggests structural changes in the cortex and reward areas that support functional changes in the response to infants.

#### 2.2.3 Endocrine changes in animals

In species that exhibit biparental care, fatherhood is associated with a number of changes in peripheral hormones throughout the course of the mate’s gestation period and the post-partum period. These changes have been extensively reviewed in previous literature [for instance, see (Bales and Saltzman, 2016; Saltzman and Ziegler, 2014; Storey and Ziegler, 2016)]. The literature on endocrine changes with fatherhood is not well integrated with the literature on neural changes; for instance, there are very few studies that incorporate hormonal manipulations with neural outcomes and vice versa. Here we summarize only the high-level patterns of change in hormonal levels as males become parents, and focus instead on connections to the neural systems involved in paternal behavior, while highlighting crucial gaps in our understanding of how these endocrine changes are functionally related to the onset and maintenance of behavior. The strongest evidence suggests that for many species, lower testosterone (perhaps associated with increases in estrogen due to conversion from testosterone), as well as central release of OT and AVP, may contribute to the neural response to infants in fathers. We therefore do not discuss some other hormones (i.e., estrogens, prolactin, glucocorticoids) for which there has been substantial research, but findings are mixed or suggest that the hormone is not critical in mammalian paternal care (Saltzman and Ziegler, 2014; Rilling et al., 2025; Chauke et al., 2011; Harris et al., 2011; Perea-Rodriguez et al., 2015).

Given that testosterone has an important role in male secondary sex development and mating and that patterns of testosterone differ between polygynous and socially monogamous species (Ketterson and Nolan, 1999), it is unsurprising that testosterone has been a focus of study for the subsequent period of paternal care for biparental species, and is the best studied hormone in human fathers. In these species, the female’s gestation period seems to be associated with an increase in testosterone immediately prior to birth, with a drastic drop-off soon after parturition [Mongolian gerbils, *Meriones unguiculatus* (Brown et al., 1995), black tufted-ear marmosets, *Callithrix kuhli* (Nunes et al., 2000), cotton-top tamarins, *Saguinus oedipus* (Ziegler et al., 2004)]. Possibly, the pre-birth increase in testosterone may facilitate male stranger aggression and protection of the gestating female, as well as readiness for post-partum mating (Nunes et al., 2000). Then, the testosterone drop following birth could subsequently reduce infanticidal behavior, though results are inconsistent in terms of how testosterone impacts infanticide (Elwood and Stolzenberg, 2020). Decreased testosterone may also promote paternal behavior, though the effect of this drop seems to vary by species, as lower testosterone results in increased paternal behavior in some species [for instance, black tufted-ear marmosets (Nunes et al., 2001)] but decreased paternal behavior in others, such as California mice, *Peromyscus californicus* (Trainor and Marler, 2001). Thus, it might be that testosterone’s role in paternal behavior comes in the form of a decrease that serves two purposes: first, in reducing infanticidal behavior, while also allowing for the onset of paternal behavior in some species, as spurred by interactions with other hormones.

In animals, peripheral OT levels generally increase in fathers or expectant fathers when compared to typical levels of naïve males [California mice (Gubernick et al., 1995)], and fathers may show increased OT immunoreactive neurons in several brain regions [prairie voles (Kenkel et al., 2014), though also see (Wang et al., 2000)]. Hypothalamic explants from common marmoset fathers also produced more OT than those from non-fathers (Woller et al., 2011); however, levels of OTR in prefrontal cortex did not change with parenthood (Kozorovitskiy et al., 2006). In titi monkeys, OTR in the presubiculum was higher in parents (Baxter et al., 2020). As mentioned in a previous section, OT neurons projecting from the amygdalohippocampal area to the MPOA inhibit infanticide in male mice (Sato et al., 2020). California mice fathers that received intranasal OT were quicker to approach pups (Guoynes and Marler, 2022). Studies of parenting in prairie voles have shown that the levels of early parenting received can affect the methylation of OTR, in ways that lead to altered social behavior (pair bonding and parenting) as an adult (Danoff et al., 2021; Perkeybile et al., 2018). However, there is still a lot of work to be done to clarify the role of OT in the neural circuitry of fathering, the relationship between peripheral and central levels, and the role (if any) of peripheral OT in male parenting.

AVP and OT have both been linked to the onset of paternal care in biparental species. However, AVP has rarely been studied peripherally in the context of fatherhood in non-human species, with most studies assessing its expression and activity in the central nervous system; for instance, there is evidence of increased AVP expression in prairie vole (Bamshad et al., 1994; Wang et al., 2000) and California mice (Bester-Meredith et al., 1999) fathers. Important exceptions include the findings, mentioned earlier in this paper, that common marmoset fathers have increased levels of AVP receptors in the prefrontal cortex, which declines as infants get older (Kozorovitskiy et al., 2006), and that titi monkey males have the opposite pattern throughout the forebrain (Baxter et al., 2023). However, prairie voles that were infused with AVP showed increased paternal behavior and an AVP antagonist inhibited paternal behavior (Wang et al., 1994); the same was true in the facultatively biparental meadow vole (*Microtus pennsylvanicus*).

It is likely that AVP and OT interact to produce the behavioral repertoire of fathers in biparental species. In prairie voles, it is only through a combined AVP/OTR antagonist mixture that paternal behavior was inhibited (Bales et al., 2004); antagonizing either AVP receptors or OTR on their own did not significantly reduce paternal huddling or pup-licking below control, suggesting that both AVP and OT are capable of facilitating aspects of paternal behavior. It may also be that AVP and OT promote synchronous affiliative behavior with both mate and pups and that these interactions, in turn, promote further expression of these hormones. However, interactions between AVP and OT in producing paternal care (and the stimuli that promote their production) need to be further empirically explored to fully characterize their effects.

#### 2.2.4 Endocrine changes in humans

Consistent with this hypothesis, human fathers-to-be demonstrate patterns of testosterone fluctuation that support a relationship between lowered testosterone and parental care. Significant decreases in testosterone were seen in pre- to post-natal comparisons (Bakermans-Kranenburg et al., 2022; Rilling et al., 2025) and were further related to higher parenting quality (Meijer et al., 2019; Rilling et al., 2025). Meta-analyses of the relationship of testosterone to fatherhood in humans concluded that the effect size of fatherhood was small to medium (Grebe et al., 2019; Meijer et al., 2019). They found that not only were pair-bonding and parenting associated with lower testosterone levels, but on an individual basis, lower testosterone levels were associated with higher quality/more involvement in pair-bonding and parenting (Grebe et al., 2019). In addition, variability in the androgen receptor is a fruitful, but so far under-studied piece of the puzzle to integrate; genetic variation in this receptor has been associated with paternal care in humans (Gettler et al., 2017; Mascaro et al., 2014; Storey et al., 2020).

Testosterone levels in human fathers have also been linked to neural activity (Abraham and Feldman, 2022). Testosterone levels are generally inversely correlated with neural responses to child stimuli, including photos (Mascaro et al., 2014) and infant cries (Khoddam et al., 2020), with some complicated interactions between fathers and non-fathers. Testosterone levels are also inversely correlated to neural activity in the ventral tegmental area, where the dopamine for the mesolimbocortical system is produced (Mascaro et al., 2013). Intervention studies are still needed to assess causal links (Abraham and Feldman, 2022).

Human fathers display lower levels of peripheral AVP than non-fathers (Rilling et al., 2025), while potentially demonstrating increased sensitivity. One study administered AVP to expectant fathers and showed that after the birth of their babies, they demonstrated less use of force when exposed to own infant stimuli versus other infants (Alyousefi-van Dijk et al., 2019). Similar findings by Cohen-Bendahan et al. (2015) found that administering vasopressin in fathers-to-be increased men’s watching time of baby-related stimuli during the third trimester of their partner’s pregnancy. These human studies would be consistent with the findings of up-regulated AVP receptor availability in the brains of common marmoset fathers (Kozorovitskiy et al., 2006).

A growing body of work has also examined plasma and salivary OT and their relationship to human fathering, as well as the results of OT manipulation in humans (Abraham and Feldman, 2022). In human fathers, OT levels are correlated with less parental touch (Feldman et al., 2012), while administration of OT to the father significantly increased male parenting behavior as well as the infants’ OT (Weisman et al., 2012). In humans, plasma OT levels increased across the first 6 months of fatherhood (Gordon et al., 2010b), a conclusion also reached in a systematic review of the literature (Grumi et al., 2021). One study integrated plasma OT, genotype of OTR, and behavior. Results suggested that peripheral and genetic markers of the extended OT pathway are interrelated and underpin core behaviors associated with human parenting and social engagement. These findings may have important implications for understanding neuropsychiatric disorders marked by early social dysfunctions (Feldman et al., 2012).

While OT expression is indeed associated with paternal behavior in many studies, OT’s known role in the maintenance of pair-bonding with the male’s mate is difficult to distinguish from its role in paternal behavior, a problem that is being increasingly recognized in the literature; for instance, a recent human study controlled for relationship quality (Rilling et al., 2025). Compounding this difficulty in disentangling why OT increases in expectant, pair-bonded fathers, manipulation of OT to determine causal effects on paternal behavior is rare. This is supported by the fact that human fathers exhibit increased parental bonding behaviors with their infants following exogenous OT administration (Weisman et al., 2014).

## 3 Effects of parenting on fathers

Given the profound nature of the changes in the brain, hormones, and experience of fathers, fatherhood’s effects on paternal health remain understudied. Existing literature focuses on how paternal health affects offspring wellbeing (Dimofski et al., 2021; Watkins et al., 2020), or alternatively, how male health behaviors affect reproductive measures such as sperm vitality (Craig et al., 2017; Engel et al., 2021; Trautman et al., 2023). Given the energetic costs of parenting, it could be expected that species displaying paternal care show physiological changes during offspring care; however, research that has followed men’s health during paternity has yielded inconsistent results, varying across both studies and species, suggesting that context and parental system might moderate how fatherhood impacts well-being.

### 3.1 Paternal physical health

#### 3.1.1 Paternal physical health in animals

Research discussing paternal health is inconsistent, and rodent work studying paternal health varies. Physiologically, prairie vole fathers have lower heart rates compared to virgin males when exposed to a novel pup (Kenkel et al., 2014). Prairie vole fathers tend to lose weight over time (Campbell et al., 2009; Kenkel et al., 2014), a pattern that differs from primates (Ziegler et al., 2006), and they have larger testes and less subcutaneous fat relative to body weight compared to virgin males (Kenkel et al., 2014). Preserving testicle size during fatherhood-related fat loss may be associated with preserving reproductive function during postpartum estrus in prairie vole females.

In California mice, differences between virgin, pair-bonded, and breeding males are variable. Zhao et al. (2017) did not find differences between groups in body mass, fat mass, or lean mass, an unexpected result given previous work showing that male California mice housed with a primigravid female have a lower body weight (Saltzman et al., 2015) and that experienced fathers gain body mass during a partner’s second pregnancy while still caring for their first litter (Harris et al., 2011; Saltzman et al., 2015). This juxtaposes patterns exhibited by other biparental mammals, in which fathers gain weight during partner pregnancy and subsequently lose it postpartum during infant dependency [reviewed in Saltzman and Ziegler (2014)]. Additionally, California mice show differences after fathering in fasted triglyceride levels, where breeding males have lower triglycerides than virgin males (Zhao et al., 2017). The biological significance of lipid metabolism changing during fatherhood is unknown, but one possibility is that lipid metabolism is influenced by gonadal steroids (Santosa and Jensen, 2015), which differ across the reproductive state (Trainor et al., 2003).

Changes in weight and energetic status across fatherhood are unclear in primates, although patterns in body mass have been established. Both common marmosets and cotton-top tamarins experience weight gain throughout partner pregnancy, followed by postpartum weight loss (Ziegler et al., 2006). While females also gain weight during pregnancy, males gain weight on different timetables to their pregnant partners; males gain weight earlier in pregnancy than females, who typically gain weight later in pregnancy during periods of fetal growth (Ziegler et al., 2006). This is hypothesized to prepare the male for the energetic costs associated with infant care, although the association between weight gain and parenting remains unknown. It is possible that weight gain may influence parental investment, affect male responses to female cues (Ziegler, 2009), or help fathers survive times of resource scarcity (Gettler et al., 2017).

#### 3.1.2 Paternal physical health in humans

In humans, weight gain as a father is a well-established pattern (Umberson et al., 2011), although the mechanisms are ultimately unknown (Saxbe et al., 2018). A study of 10,000 men showed that fatherhood predicted increased BMI, while childless men showed decreased BMI over the same time period (Garfield et al., 2016). With the attentional, emotional, and physical challenges of parenthood, weight gain could be moderated by sleep disruptions, increased stress, and/or decreased physical activity (Saxbe et al., 2018). It is unclear how this weight gain may affect men in the long term and if this weight gain places fathers at a higher risk for disease.

Although a majority of adult men are fathers (Monte and Knop, 2019), there is currently no established population-based system for collecting health data from men in the perinatal period [noted by Garfield et al. (2022)]. Studies that have delved into these questions show varying results. For example, Gjerdingen and Center (2003) found that fathers experience more sick days and decreased health and vitality, while Asselmann et al. (2022) found no significant, meaningful changes in men’s physical health in response to their first child. These differences could result from differential gender roles and expectations for men and women. Some studies have shown that paternal alcohol use declines postpartum (Torche and Rauf, 2021), while others suggest that the rate of alcohol and tobacco use is not largely different in fathers compared to the general population (Garfield et al., 2022). Although some studies show that fathers display healthier habits, other studies suggest their health declines during fatherhood, which is a phenomenon that needs to be further explored given the conflicting results.

Paternal investment does not seem to largely affect long-term male health. A 15-year study found that time spent with one’s children, satisfaction with being a parent, and the degree to which an individual worried about their children did not affect a man’s risk of death, heart disease, stroke, or cancer (Hibbard and Pope, 1993). A smaller study found that men with child custody and men with stable relationships had fewer medical issues than those living alone (Hallberg, 1992), although these results could be the result of a stable homelife, rather than parenting itself.

No work to date has holistically followed fathers throughout their lifespan to establish how physiology changes during parenting and, subsequently, how those changes affect parenting techniques or offspring attachment, as parenting is expected to fluctuate throughout their offsprings’ developmental trajectories. Future work would benefit from addressing how men’s physiology changes during gestation and parenthood while accounting for individual differences in health behaviors, and by evaluating how changing health affects parenting behaviors, parental experience, and infant attachment.

### 3.2 Paternal mental health

Fatherhood is commonly identified as starting at birth (Scism and Cobb, 2017), a period during which fathers are at risk of paternal postpartum depression [PPPD (Pilyoung Kim and Swain, 2007)]. Experiencing postpartum depression can heavily impact a father’s ability to provide care for his children (Chen et al., 2023; Sethna et al., 2015; Wells and Jeon, 2023). However, despite a prevalence of PPPD reported up to 25% in fathers (Chavis, 2022; Kim and Swain, 2007), most postpartum depression work lies in the understanding of maternal risk factors. Known PPPD risk factors include mother’s postpartum depression onset of symptoms, the father’s relationship with his own parents, unemployment, negative life events, relationship dissatisfaction, and the father’s previous experiences with depression prior to entering parenthood, etc. (Areias et al., 1996; Matthey et al., 2000; Ansari et al., 2021; Wang et al., 2021; Sokół-Szawłowska, 2020).

Current theories in maternal postpartum depression stem from low levels of brain-derived neurotrophic factor (BNDF) following birth, but similar findings are not found for males (Kittel-Schneider et al., 2022). An emerging rat model of postpartum depression supports the theory that a mother’s onset of postpartum depressive symptoms is a risk factor for depressive symptoms in paternal partners while also showing a higher rate of apoptotic neurons in the hippocampus (Kocahan et al., 2021). However, first-time fathers with positive father-child bonds but without known depression have increased left hippocampal volume, showing the hippocampus may be important in the preparation for fatherhood (Saxbe et al., 2023) and impairment may increase the risk for PPPD. Hormonal differences may also be driving PPPD; months before birth, a father’s testosterone levels drop and the risk for depressive symptoms increases (Kim and Swain, 2007). When testosterone levels stay elevated, fathers are less likely to be depressed, but the bonds formed both with partners and offspring are impaired, resulting in more violent behaviors (Saxbe et al., 2017b).

Within the family, PPPD can result in detrimental interpersonal behaviors and bonds. Hippocampal changes between the prenatal and postnatal period correlate with differences in parent–child bonds, such that reductions in hippocampal volume result in impaired bonds (Saxbe et al., 2023). Fathers suffering from postpartum depression engage differently with their offspring: less gentle touch, less active engagement, and less playful excitation play (Sethna et al., 2018). Postpartum-depressed fathers were more withdrawn, displaying less verbal and behavioral stimulation when interacting with young infants (Sethna et al., 2015). PPPD has also been associated with increased rates of fathers’ physical violence towards their children (Saxbe et al., 2017b) and a higher risk of violent behaviors toward their partners - one in four women report abuse from first-time fathers, and 69% of such cases represent the first incidence of violence in the relationship (Kim and Swain, 2007).

Interventions are needed to identify significant differences in the behavior of fathers suffering from postpartum depression (Kim and Swain, 2007; Sokół-Szawłowska, 2020; Wells and Jeon, 2023). A recent systematic review found only 14 peer-reviewed publications, covering 10 interventions, for PPPD (Goldstein et al., 2020). Maintaining a positive co-parenting relationship can help mediate the risk of PPPD (Wells and Jeon, 2023). A case study showed treating PPPD similarly to other mood disorders alleviates the negative consequences associated with paternal parenting (Sokół-Szawłowska, 2020). In addition, when fathers were educated to detect postpartum depression, they were more likely to identify symptoms in themselves (Guillemette et al., 2023), emphasizing the need for more educational training; especially as male depressive symptoms differ from females (Brownhill et al., 2005; Scarff, 2019). Fathers report needing a longer duration to feel connected to the infant with a range of 6 weeks to 2 months (Scism and Cobb, 2017), but once fathers have bonded with their infants, they report high levels of satisfaction and a large sense of connection (Goodman, 2005), which may mediate the correlation to PPPD (Wells and Jeon, 2023).

PPPD has been identified as a significant factor influencing parenting behaviors and father-child relationships. A meta-analysis by Wilson and Durbin (2010) revealed that paternal depression was associated with decreased positive parenting and increased negative parenting behaviors, such as hostility and disengagement. Similarly, research suggests that depressed fathers are less likely to read regularly to their one-year-old children and are more likely to report spanking compared to non-depressed fathers (Davis et al., 2011). Another study similarly demonstrated that paternal depression correlates with reduced engagement and positivity in father-infant interactions, which predicts greater behavioral issues in young children (Barker et al., 2017).

### 3.3 Paternal cognitive health

#### 3.3.1 Paternal cognitive health in animals

Gestation, birth, and parental care have been shown to impact cognitive function in female parents as compared to virgin mother rodents [though stress may moderate some of these effects, as reviewed in (Pawluski et al., 2016)]. Presumably, such changes are related to increased neuronal plasticity in key brain regions, such as the hippocampus and prefrontal cortex. California mouse fathers showed increased spatial learning in a dry-land maze task and showed increased biomarkers of hippocampal neuroplasticity (Franssen et al., 2011), mirroring work suggesting similar changes in mothers. However, in contrast, California mouse fathers did not show differences in object recognition or exploration tasks (which reflect short-term memory and novelty-seeking behavior, respectively) as opposed to virgins (Glasper et al., 2011).

#### 3.3.2 Paternal cognitive health in humans

Parenthood in humans appears to modestly reduce dementia risk, with similar effect sizes in men and women (Basit et al., 2023). A study of left hippocampal volume in fathers who were scanned twice, once before the birth of their child and once 6–8 months after the birth, found no difference in hippocampal volume. However, larger volume increases were positively correlated with adaptation to parenting, and higher prenatal OT predicted greater positive change in hippocampal volume from pre- to post-natal (Saxbe et al., 2023). Although human fathers may show increased neuroplasticity in similar regions as mothers (Horrell et al., 2021), there has been little exploration of how fatherhood changes performance on cognitive tasks, especially when controlling for stress-related deficits in cognition.

## 4 Fathers’ interactions with others

### 4.1 Partner relations and paternal care

#### 4.1.1 Partner relations and paternal care in animals

Becoming a parent involves shifts in partner dynamics in preparation for the baby, upon the baby’s arrival, and during coordination of caretaking responsibilities in addition to maintaining a quality relationship as a couple. In socially monogamous, biparental titi monkeys, pairs in which males spent more time carrying the infant exhibited lower affiliation between the parents; however, affiliation rates in these same pairs rebounded sooner as compared to pairs with average- or below-average male carry rates, suggesting that higher paternal care may serve to facilitate post-partum affiliation (Karaskiewicz et al., 2021). Another sample of adult titi monkeys found that, for parenting individuals, average levels of partner affiliation were strongly correlated with OTR binding in layer 1 and layer 3 of the presubiculum as well as the total presubiculum, though OTR binding was negatively correlated with proximity and contact behavior with the partner (Baxter et al., 2020).

#### 4.1.2 Partner relations and paternal care in humans

In humans, quality marital and close relationships reduce the risk of disease and improve recovery from illness (Kiecolt-Glaser, 2018; Kiecolt-Glaser and Newton, 2001). The transition to parenthood is a significant life event for couples that can shift their relational dynamics and qualities. For example, from pre- to post-birth, fathers reported significant gradual decreases in relationship confidence and steady increases in problem intensity after birth, but about only 15% of the fathers in the sample reported improvement in overall relationship satisfaction during the transition to parenthood (Doss et al., 2009). Indeed, these aspects of positive interparental relationships during fatherhood have a dearth of, yet burgeoning, empirical evidence in human and non-human animal literature.

There is correlational evidence that hormone levels may be associated with pre- and post-partum relationship quality. One study found that for fathers who have higher prenatal testosterone levels, their partners reported lower relationship quality during the postpartum period (Cárdenas et al., 2023). Relatedly, greater decline in fathers’ testosterone across pregnancy is associated with higher levels of mothers’ postpartum investment and satisfaction (Saxbe et al., 2017b). Fathers showing larger prenatal testosterone declines were rated as providing more postpartum support by their partners, though this slope was not moderated by fathers’ own perceptions of perceived support from their partners (Edelstein et al., 2017). Correspondingly, mothers showing smaller testosterone increases throughout the prenatal period were rated as providing higher spousal support by their male partners (Edelstein et al., 2017). The same direction of effects was found with estradiol, with larger paternal declines and smaller maternal increases in estradiol during the prenatal period predicting higher partner-rated spousal support postnatally (Edelstein et al., 2017). When undergoing a parent-infant attachment paradigm, mothers and fathers showed comparable declines in testosterone, with fathers who had a more avoidant attachment orientation showing greater attenuation (Edelstein et al., 2017). A recent systematic review found that the highest evidence for physiological synchrony between parents was for prenatal cortisol (Daneshnia et al., 2024).

“Synchrony,” within the social behavior literature, carries a nebulous definition—it’s often referred to as attunement, linkage, coordination, mutuality, reciprocity, rhythmicity, among many other terms. For our purposes, we will utilize the definition provided by Feldman (2017). Here, synchrony is defined as “the coordination of biological and behavioral processes between attachment partners during social contact.” We emphasize temporal alignment as a key factor. For example, a caretaker smiling because they noticed their infant smiling would be a behavior that is temporally aligned and responsive/contingent, and therefore synchronous. Physiological synchrony between parenting partners may serve to maintain the relationship throughout child-rearing. Here we describe physiology synchrony as intercorrelations between partners’ physiological patterns in a given period (and not contingent responses to partners’ physiological reactivity). Some evidence of spousal synchrony (within-couple associations; intercorrelations within physiological patterns) in testosterone levels has been linked to positive relational outcomes. Greater prenatal testosterone synchrony predicted higher levels of postpartum relationship quality for fathers and a decrease in fathers’ testosterone from prenatal to postpartum (Cárdenas et al., 2023). Although cross-sectionally, prenatal and postpartum spousal testosterone synchrony were not associated with fathers’ reports of couple relationship quality, longitudinally, stronger spousal testosterone synchrony during pregnancy predicted fathers’ reports of better couple relationship quality postpartum (Cárdenas et al., 2023). Uniquely, prenatal testosterone synchrony predicted higher levels of father-reported postpartum investment, commitment, and satisfaction with their partners, but not for mothers (Saxbe et al., 2017b).

Other work has also explored spousal synchrony in the context of parenthood for physiological and neuroimaging outcomes. During a supportive interaction, first-time expectant parents exhibited positive heart rate synchronization with each other (Fogel-Yaakobi et al., 2023). Higher avoidant attachment was in general associated with higher cardiac synchrony among the expectant parents in the sample (Fogel-Yaakobi et al., 2023). Further, for those with higher levels of anxious attachment, cardiac synchrony with their partners was associated with less supportive behavior; conversely, for those with lower levels of attachment anxiety, cardiac synchrony was not significantly associated with supportive behavior (Fogel-Yaakobi et al., 2023). During conflict interactions, when presented with infant stimuli, fathers reacted with sympathetic vigilance by preserving sympathetic arousal, but this pattern was not observed with mothers (Mosek-Eilon et al., 2013).

### 4.2 Father-child synchrony

This section focuses on humans only, because there is a lack of data on father-infant synchrony in non-human animals. Behavioral synchronization is thought to be fundamental to a child’s social and cognitive development (Ruth Feldman, 2012), and evidence has shown consistently that greater parent–child behavioral synchrony is associated with greater youth self-regulation (Davis et al., 2017). In studies of behavioral synchronization in parents and infants, synchrony is often indexed by the probability of the parent making a coordinated behavior in response to infant behavior, such as returning infant gaze, reflecting positive infant affect, vocalizing following infant vocalization, and returning affectionate touch (Atzil et al., 2014). Feldman (2003) used a similar criterion for synchronization, finding that there was a distinct infant arousal pattern during father-infant interactions, consisting of several bursts of emotional intensity that seemed to appear at random and could be reached from any initial state. This was much different from mother-infant interactions, which showed cyclical oscillations between low and medium infant states of arousal. These findings are consistent with the idea that mothers tend to engage in more social behaviors with their infants and express greater emotional reciprocity when interacting with their children (Thomassin and Suveg, 2014), while fathers tend to engage in physical play more than social play (Amodia-Bidakowska et al., 2020; MacDonald and Parke, 1986). Subsequently, fathers’ patterns of behavioral synchrony differ from mothers’. It is important to note however, that higher arousal does not necessarily result in higher synchrony. In instances in which there is less affective matching (for instance, a child who is more subdued in personality with a father who interacts with them with these sudden high arousal bursts), there will be less overall synchrony. Findings have indicated that this may lend to greater synchrony in same-gender parent–child dyads, thanks to greater affective matching (Feldman, 2003). Another study found that in play-specific settings, mothers and fathers express similar levels of effort in engaging their child, but mothers achieved greater behavioral synchrony (Bureau et al., 2014).

Contemporary research notes that less synchronization does not necessarily equate with negative parenting outcomes. In a recent study, researchers aimed to understand differences in inter-subject synchronization between mother–child and father-child interactions during shared experiences (Liu et al., 2024). They found that during play interactions, mothers showed more controlling behaviors, which may be due to heightened attunement and synchronization with the child’s state. Within mother–child pairs, there was greater evidence for joint gaze and joint imitation play with children, whereas fathers spent more time gazing at other things. However, fathers scored higher on sensitivity, non-intrusiveness, and non-hostility than mothers. This suggests that fathers are less controlling over their child’s play behaviors, which may be beneficial for the development of other cognitive abilities, such as independence and free play.

Physiological synchrony, while still leaning into aspects of co-occurrence and coordination, tends to be a bit more abstract in the sense that it is largely unconscious processes or actions that are coordinating (which may be more difficult to measure and interpret, especially when taking into account temporality). Often, this flavor of synchrony manifests in the literature as a sort of “physiological linkage” (Saxbe et al., 2017a), or coregulation/covariation of some state or condition. While the coordinating aspects of processes in social contexts described by Feldman (2003) remain relevant, it is also good to be aware of how this may be more broadly interpreted (i.e., with the coordination of particular physiological processes being categorized as synchrony, with these physiological processes being viewed as predictors of behavioral synchrony, etc.).

Cortisol synchrony, or adrenocortical attunement, offers a rich realm of exploration in the context of parent–child dyads. Research centering on father-child dyads, however, remains exceptionally limited. To date, only work by Saxbe et al. (2017a) has specifically explored father-child dyadic cortisol synchrony. Results indicate that cortisol synchrony was higher for father-preschooler dyads in which fathers had lower sensitivity and observed reciprocity. Looking at broader triadic synchrony, it appears that only among mothers is cortisol associated with lower levels of family synchrony (Gordon et al., 2010b). In light of our interests in father-child synchrony, it becomes increasingly clear that not only must we consider contextual factors that are highlighted in mother–child adrenocortical attunement, but also make note of how the unique role of fatherhood may enrich and expand upon these findings.

Similar to the predominantly mother-centric literature involving parent–child synchrony, OT levels can be valuable in investigating father-child interactions. Fathers have been shown to have significantly greater levels of plasma OT than non-fathers (Mascaro et al., 2014) but expecting fathers and non-fathers did not differ in their OT levels (Cohen-Bendahan et al., 2015). This suggests that OT concentrations may be more relevant in contexts of paternal care itself as opposed to during pregnancy, as is the case with mothers (Borrow and Cameron, 2012). Work by Gordon et al. (2010a, 2010b) demonstrated that parental OT levels did not vary between mothers and fathers across a study period of 6 months (assessed at the first postpartum weeks and 6 months postpartum) and that both paternal and maternal OT were able to positively predict triadic synchrony, which refers to the coordination of factors like physical proximity, touch, and social gazing between parents as well as the infant (three individuals). However, levels of plasma and salivary oxytocin have been found to relate to mother-infant and father-infant-specific interactions, characterized by an emphasis on affectionate touch for mothers or stimulatory play for fathers (Feldman et al., 2010; Gordon et al., 2010b). This is further supported by a more recent study that addresses the issue of extracted vs. non-extracted samples, a controversy within the methodological literature, by demonstrating that fathers’ playful proprioceptive touch is associated with both extracted and unextracted OT (Morris et al., 2021). Further investigation of the role of oxytocin in father-child synchrony is needed to better understand this important interaction.

Respiratory sinus arrhythmia (RSA) refers to the normal variability in heart rate that is associated with breathing, which can serve as an indicator of self-regulatory capacity. Work by Lunkenheimer et al. (2021) suggests differences between mothers and fathers in their manifestations of parent–child RSA synchrony (related changes in RSA in either the positive or negative direction), namely in its key moderators: in fathers, this included greater externalizing behaviors and positive dyadic affect, whereas in mothers, this included greater externalizing behaviors and child average RSA. This suggests potential similarities in risk assessment to RSA dyadic synchrony, albeit with more emphasis on observable states for fathers and attunement to child regulatory capacities for mothers. Differences between fathers and mothers are also noted by Fuchs et al. (2021), in which significant associations between father and child RSA (child-to-father and father-to-child) were only observed in cases where fathers’ psychological distress was higher, whereas with mothers, psychological distress could be higher or lower.

Measuring neural synchrony between parent–child pairs typically consists of observing patterns of synchronized brain activation. Contemporary work often makes use of hyper-scanning techniques whereby brain activity is measured simultaneously in both members of a dyad, making it ideal for high temporal resolution measures of the interpersonal synchronization of brain activities (Nguyen et al., 2020). The state of the literature regarding father-child neural synchronization seems to support the idea that fathers and mothers differ in their patterns of synchrony with offspring. While the variation in parent–child brain synchronizations between fathers and mothers could be attributed to several factors, the pattern aligns with behavioral research showing that fathers generally engage in different modalities of play than mothers. This may be due to a primary/secondary caretaker effect, in which fathers typically take on a secondary caretaker role (Liu et al., 2023), and therefore may not necessarily need to be responsible for sustaining low-neutral arousal states in the infant. However, the existence of these different modalities may still be essential for the development of different cognitive and social abilities in infants and children.

## 5 Discussion, limitations, future directions, and gaps in the literature

Within both the human and non-human animal literature, the existing body of research has focused so profoundly on motherhood, such that holistic investigations on fatherhood have not yet reached a depth and scope comparable to the work done with mothers. As a result, the extant work on fathering over time primarily situates itself in 1) the transitional period from non-fathering male to the onset of childbirth and the immediate developmental range of early infancy (i.e:, becoming a father), and 2) the social ramifications of including fathering/fatherhood/fathers in conversations of parenting and child development (i.e., perceptions of men as fathers). As a result, the nascent field has yet to catch up to extending the conversation to the level of biological mechanistic exploration (beyond the perinatal period) or much-needed longitudinal work, and many gaps remain.

In the review above, we summarized the current literature on fathering, including the neural and hormonal shift from non-father to father, the effects of fatherhood on father’s physical, mental, and cognitive health, and the effects on father’s relationships with the partner. Finally, we discussed the father-child relationship, especially with regard to father-child synchrony. The literature reviewed supports the central role of the MPOA and reward circuitry in the transition to fatherhood and suggests that AVP receptors across the forebrain are involved, as well as a decrease in testosterone. Once a father, experience with the infant leads to both structural and functional changes in the brain, including loss of gray matter, particularly in cortical areas; however, functional patterns may vary based on whether the father was the primary or secondary caregiver. Oxytocin is also important, possibly as a way of synchronizing father-child interaction. Finally, fatherhood may lead to other consequences for the father, particularly weight gain or loss, and potentially PPPD. There can be significant effects on aspects of other relationships, such as partner synchrony. However, there are a number of limitations due to gaps in the current literature.

On the biological level, there are multiple gaps in our knowledge of the role that hormones play in fatherhood. First, the functional significance of these hormonal changes as it relates to the neural substrates, and the subsequent paternal behavior is not always clear. While for the most likely hormonal candidates we have tried to both describe changes and highlight potential behavioral effects, it is clear that the causal relationship between hormonal fluctuation and behavior is context- and sometimes species-specific. Thus, characterizing causal behavioral effects of these paternal hormone fluctuations would make the evolution of paternal care clearer. There is also a lack of understanding of how bidirectional bond quality between father and offspring might be related to these endocrine and neural changes i.e., do more affiliative and attentive fathers show a difference in the strength of the relationship with their offspring as measured from both the father’s perspective *and* the offspring’s perspective? This bond quality is an important consideration in studies of human fathers, but has been largely ignored in non-human species, with a few notable exceptions [such as recent studies in titi monkeys, *Plecturocebus cupreus* (Witczak et al., 2023, 2025)]. Thus, a key area of expansion of our understanding is in how the bidirectional relationship between father and offspring might influence later neural, behavioral, and health outcomes. One other such area is in a more specific understanding of the relationship between peripheral hormone levels and causal changes in the brain or in fathering behaviors. Finally, transcriptomics results continue to suggest additional targets that may be important in the neurobiology of fathering (Seelke et al., 2018; Duclot et al., 2022; Rogers et al., 2026).

As suggested above, the work on the neural basis of human fathering has been limited to fMRI and peripheral hormones. While there are clear ethical reasons to focus on these non-invasive methodologies, there are other possibilities that have largely been neglected so far, and which would lend themselves better to exploring the neurochemistry of human parenting. These possibilities include PET scanning for specific receptor types, for example, as in studies of the relationship of dopamine to drug and alcohol use (Shokri-Kojori et al., 2021; Chumin et al., 2024; Jangard et al., 2023). Studies such as these would allow us to test assumptions, for instance that activity in “dopaminergic areas” is actually dopamine activity. Another possibility for more precisely studying neurochemical changes in human fathers is to access postmortem tissue banks. Human brain tissue is collected under different circumstances. However, titi monkey brain collected opportunistically from animals euthanized for a variety of health reasons, still showed large differences in V1a binding between parents and non-parents (Baxter et al., 2023). Tissue collected at different intervals postmortem showed negligible differences in at least one study of receptor binding (Freeman et al., 2017).

While species and methodology differences continue to enrich the literature on fatherhood, they also make it more difficult to interpret. For instance, the impacts of fatherhood on cognition seem to be process- and task-dependent in at least one species, but this knowledge gap means that broad generalizations and comparison to the changes of motherhood are, currently, nearly impossible. The studies of human neural changes with fatherhood use different timepoints and different tasks with different infant stimuli. On top of this, the animal and human literature often do not inform each other or allow for direct comparisons. Jointly designed studies (animal/human), with a strong emphasis on validated methodologies, timepoints, and outcome measures, could be very valuable.

Further, examining the links between paternal relationships with partners and paternal behavior is important in understanding the health and well-being of fathers, children, and the family unit. For example, it is clear that PPPD has vast implications on the health of the father and his ability to provide care (Sethna et al., 2015). There is a dire need for a consistent diagnosis designed for fathers (Kim and Swain, 2007), this would allow healthcare professionals a clearer map for detecting PPPD (Essadek et al., 2024). Using questionnaires originally designed for detecting maternal postpartum depression can limit how well PPPD is identified, where questionnaires may instead be detecting distress rather than depressive symptoms (Massoudi et al., 2013). In addition, considering cultural norms and differences may impact the PPPD diagnosis rate; for example, the Latinx community is comprised of multiple cultural norms which dictate a father’s involvement and how they manage stressors; they also have low levels of seeking mental health treatment (Parchment et al., 2024). In addition, there is a need for an animal model of paternal postpartum depression. Through an animal model, we can investigate which biological changes occur in fathers to make them most susceptible.

Human studies of fathers generally contain small sample sizes from discrete sample populations, so there is a need for larger sample sizes with diverse populations. There is also a lack of human subject research exploring the diversity of human parenting and family structures outside of cisgender and heterosexual relationships, such as same-sex male couples, same-sex female couples, genderqueer parent(s), and so on. Potential intergenerational transmission of paternal care could also be explored further, as well as teasing out the effects of the environment vs. genetics.

The science of fathering, while under-studied in the past, is now coming into its own. This both reflects a change in the role of fathers in many Western cultures (Hrdy, 2024), as well as lending hope for a better understanding of the experience of fathering from many perspectives.

## Glossary

AHi: amygdalohippocampal area
AOB: accessory olfactory bulb
AVP: vasopressin
BNSTrh: rhomboid nucleus of the bed nucleus of the stria terminalis
BDNF: brain-derived neurotrophic factor
Calcr: calcitonin receptor
cMPOA: central medial preoptic area
GABA: gamma-aminobutyric acid
Gal: galanin receptor
LHA: lateral hypothalamus
MeApd: posterodorsal region of the medial amygdala
mPFC: medial prefrontal cortex
MPOA: Medial preoptic area
OT: oxytocin
OTR: oxytocin receptor
PAG: periaqueductal gray
PeFA: perifornical area of the hypothalamus
PPPD: paternal postpartum depression
PVH: paraventricular nucleus of the hypothalamus
RSA: respiratory sinus arrhythmia
STS: superior temporal sulcus
UCN3: urocortin-3
V1a: vasopressin type 1a receptors
VNO: vomeronasal organ
